# Using Feasibility Data and Codesign to Refine a Group-Based Health Literacy Intervention for New Parents

**DOI:** 10.3928/24748307-20210911-01

**Published:** 2021-10

**Authors:** Danielle M. Muscat, Julie Ayre, Don Nutbeam, Anne Harris, Lynette Tunchon, Dipti Zachariah, Kirsten J. McCaffery

## Abstract

Few health literacy interventions exist for new parents. We developed a group-based health literacy program (“Parenting Plus”), which was initially tested in a feasibility study in 2018. In this brief report, we describe how feasibility findings were incorporated into the Parenting Plus program. Using a codesign process with patient partners (feasibility study participants) and health staff to revise the program, version 2 was tested in a single-site pilot using pre- and post-intervention testing. Parents older than age 16 years whose child was between ages 4 and 26 weeks were recruited from nurse home visits in western Sydney, Australia. Interested participants attended the free 4-week health literacy program (four 2-hour sessions) delivered by a trained facilitator. Piloting suggested the revised program is acceptable to new parents, has good retention (93% over the course of 4 weeks), and can improve health literacy skills, including access to reliable health information and services. Our iterative development and codesign approach integrated learnings from various sources to inform the design of an evidence-based health literacy intervention. We now move to an effectiveness implementation hybrid trial to test intervention effectiveness (health literacy skill development) and support translation of research findings into routine practice. **[*HLRP: Health Literacy Research and Practice*. 2021;5(4):e276–e282.]**

A significant proportion of parents of newborn infants have low health literacy (Mackley et al., 2016), and a growing body of evidence shows that low parental health literacy is associated with poorer child health outcomes (Dallacker et al., 2016; Keim-Malpass et al., 2015) and greater incidence of child emergency department visits and hospitalizations (DeWalt et al., 2007). However, few interventions exist that develop health literacy skills for new parents to help them access, understand, appraise, and act on health information.

In July 2020, we reported on a study that assessed the feasibility of embedding a group-based health literacy skills training program (Parenting Plus, version 1) within established parenting groups in New South Wales (NSW), Australia (Muscat et al., 2020). Findings indicated that staff and parents wanted more information about parenting topics (e.g., starting solid foods) in addition to skills training, more time for group discussion, and content adaptable to parents from diverse backgrounds. Staff also requested greater institutional alignment by including routinely used health promotion resources.

This article should be read in conjunction with our feasibility article (Muscat et al., 2020) as we now detail how the revised version of the program was developed. First, we used the feasibility findings and a codesign process involving patient partners (new parents) and health staff (including staff who work directly with new parents and managerial staff who manage child and family health services) to develop version 2. This was then tested in a small pilot ahead of a larger randomized trial.

## Methods

### Codesign Process

Codesign involves the active contribution of patients and other health care stakeholders in the design of interventions (Sanders & Stappers, 2008; Realpe & Wallace, 2010). Our codesign team consisted of health literacy researchers, patient partners (new parents who participated in the feasibility study), and health staff (staff working directly with new parents, and managers of child and family health services). Researchers and health staff worked together to analyze and interpret feasibility study data and identify necessary modifications to program content. To facilitate this, a child and family health nurse (A.H.) was formally subcontracted in partnership with the local health district to work collaboratively with researchers over a 10-month period to revise the program. Patient partners (*n* = 3) were interviewed about their expectations for parenting programs and provided feedback on written and audio-visual materials used in the revised program. Managerial health staff iteratively reviewed program content in a series of staff workshops and follow-up correspondence.

### Pilot Testing

We conducted a single-site pilot to pragmatically test version 2 of the program ahead of a larger randomized trial. The study was approved by the Sydney Local Health District Human Research Ethics committee (protocol number HREC/17/RPAH/466).

Eligible parents were residents of western Sydney older than age 16 years with a child between ages 4 and 26 weeks; parents also needed to have sufficient English fluency to complete the program in English. They were recruited during a free home nurse visit provided to all parents in NSW. Interested participants attended the free 4-week health literacy program (four 2-hour sessions).

Using a mixed methods approach, we assessed core feasibility outcomes (Bowen et al., 2009) at baseline and immediate follow-up including (1) demand/retention; (2) acceptability; and (3) impact on short-term health literacy outcomes. For quantitative data, we calculated means and frequencies using Microsoft Excel software. All qualitative data (focus groups, observations) were analyzed using thematic analysis.

## Results

### Codesigned Program Content

**Figure 1.**
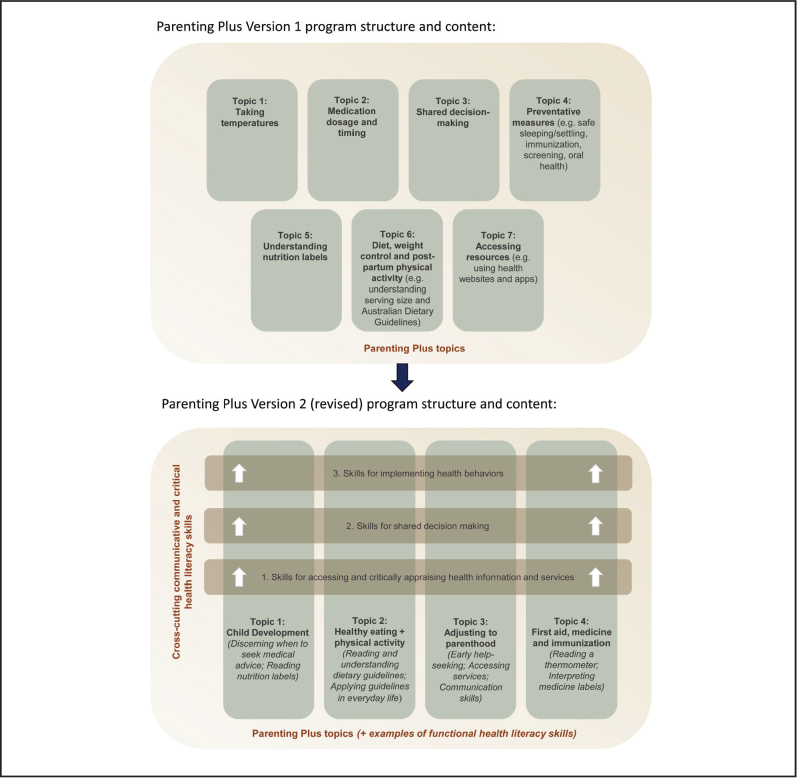
Original and revised Parenting Plus program structure and content.

Findings from feasibility testing (Muscat et al., 2020) were incorporated into version 2 of the Parenting Plus program in collaboration with health care staff and patient partners. The codesign process identified patient and staff priorities for revision and ensured modifications adequately addressed underlying issues. For example, a key revision was to embed health literacy skills into parenting topics of interest. These include skills for shared decision-making, accessing and critically appraising health resources and acting on health information (i.e., behavioral implementation) (**Figure 1**; **Figure A**). Feedback from patient partners and health staff ensured revisions achieved an acceptable balance between health literacy skills and content-specific health knowledge. For example, the revised program emphasizes how and where to access reliable Australian health information and services. This approach provides participants with the skills to independently seek out additional content that could not be covered in detail in the program.

**Figure A.**
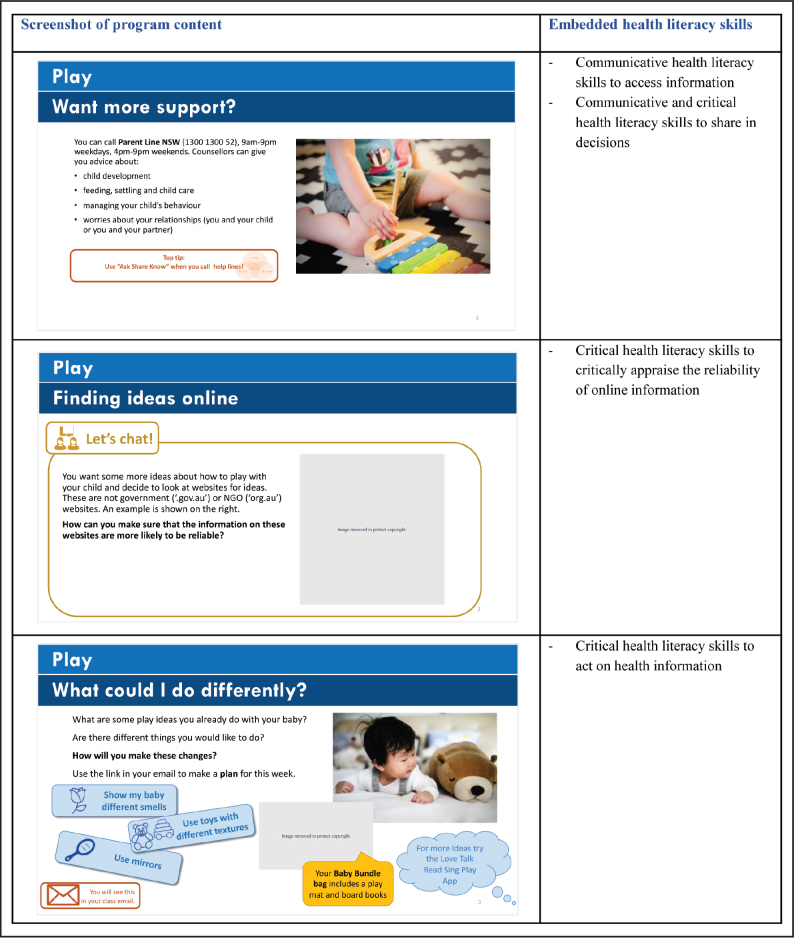
Examples of health literacy skills embedded within a broader topic of child development.

Another key revision was a shift to a more informal, interactive format. This was achieved by replacing workbooks with PowerPoint presentations, incorporating more time for discussion, and reviewing and reflecting on content from the previous week. Through codesign, discussion activities were aligned with the model of care used by child and family health nurses. We developed open-ended questions to elicit personal reflections and ensure greater capacity to engage parents with wide-ranging health literacy skills and knowledge.

### Pilot Testing

Fourteen participants attended the first session and completed baseline data, of which 13 (93%) attended all or the majority (3 of 4) of the sessions and completed the follow-up questionnaire. This represents a 31% increase in retention from the first feasibility study. The average age for participants was 33.8 years (*SD* = 4.2 years) and, the average age for infants was 9.8 weeks (*SD* = 4.1 weeks). Most participants were born in Australia (*n* = 9) and 21% had inadequate health literacy as assessed by a single-item literacy screener.

Using a purpose-designed validated parenting health literacy measure (the Parenting Plus Skills Index; Ayre et al., 2020), we observed a 1.3-point increase (out of 13) in health literacy skills from baseline to follow-up (Cohen's *d* = 0.5, representing a moderate effect size (Cohen, 2013). Using the Health Literacy Questionnaire (Osborne et al., 2013), increases were observed for 5 of 9 scales, with the largest increases on “ability to find good health information” (+0.3 on a 5-point scale) and “understand health information well enough to know what to do” (+0.5 on a 5-point scale) after 4 weeks. Parental awareness and use (within the past 4 weeks) of reliable health services and information sources increased overall (**Table 1**). Participants were most likely to share information from the program with their partner (42%), but also reported distributing information to their mother, mother-in-law, siblings, father, friends, and other parents.

**Table 1:**
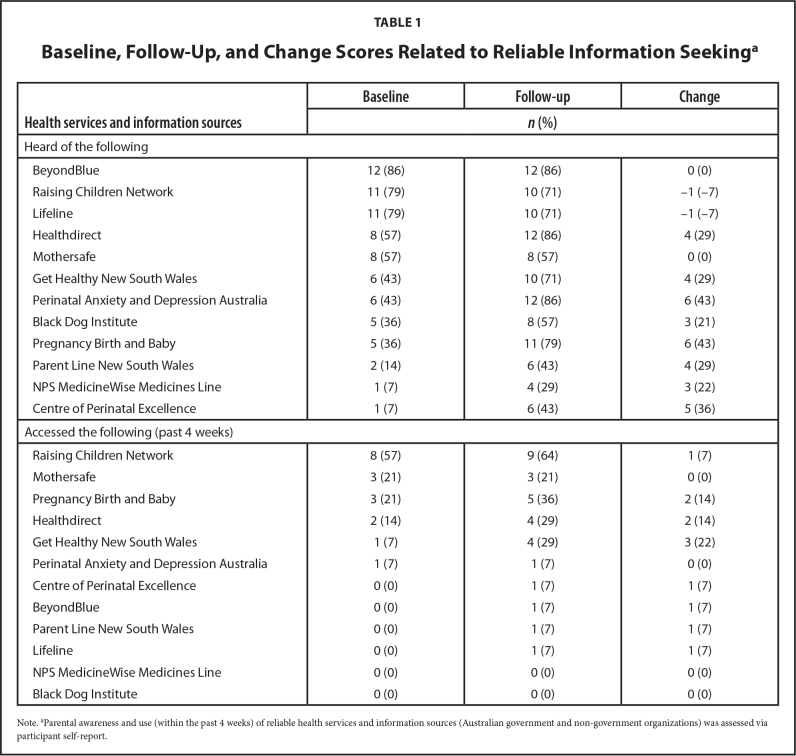
Baseline, Follow-Up, and Change Scores Related to Reliable Information Seeking^a^

**Health services and information sources**	**Baseline**	**Follow-up**	**Change**

	***n* (%)**		

Heard of the following			

BeyondBlue	12 (86)	12 (86)	0 (0)
Raising Children Network	11 (79)	10 (71)	−1 (–7)
Lifeline	11 (79)	10 (71)	−1 (–7)
Healthdirect	8 (57)	12 (86)	4 (29)
Mothersafe	8 (57)	8 (57)	0 (0)
Get Healthy New South Wales	6 (43)	10 (71)	4 (29)
Perinatal Anxiety and Depression Australia	6 (43)	12 (86)	6 (43)
Black Dog Institute	5 (36)	8 (57)	3 (21)
Pregnancy Birth and Baby	5 (36)	11 (79)	6 (43)
Parent Line New South Wales	2 (14)	6 (43)	4 (29)
NPS MedicineWise Medicines Line	1 (7)	4 (29)	3 (22)
Centre of Perinatal Excellence	1 (7)	6 (43)	5 (36)

Accessed the following (past 4 weeks)			

Raising Children Network	8 (57)	9 (64)	1 (7)
Mothersafe	3 (21)	3 (21)	0 (0)
Pregnancy Birth and Baby	3 (21)	5 (36)	2 (14)
Healthdirect	2 (14)	4 (29)	2 (14)
Get Healthy New South Wales	1 (7)	4 (29)	3 (22)
Perinatal Anxiety and Depression Australia	1 (7)	1 (7)	0 (0)
Centre of Perinatal Excellence	0 (0)	1 (7)	1 (7)
BeyondBlue	0 (0)	1 (7)	1 (7)
Parent Line New South Wales	0 (0)	1 (7)	1 (7)
Lifeline	0 (0)	1 (7)	1 (7)
NPS MedicineWise Medicines Line	0 (0)	0 (0)	0 (0)
Black Dog Institute	0 (0)	0 (0)	0 (0)

Note.

a Parental awareness and use (within the past 4 weeks) of reliable health services and information sources (Australian government and non-government organizations) was assessed via participant self-report.

Analysis of focus group data (**Table A)** indicated that participants thought the topics were relevant and interesting. They particularly valued the opportunity to socialize through group discussions, and reported feeling more aware of available resources (e.g., helplines) to support them in their parenting journey. Participants also described the usefulness of critical health literacy skills, such as evaluating the reliability of websites, engaging in shared decision-making, and implementing health changes through planning activities.

**Table A:**
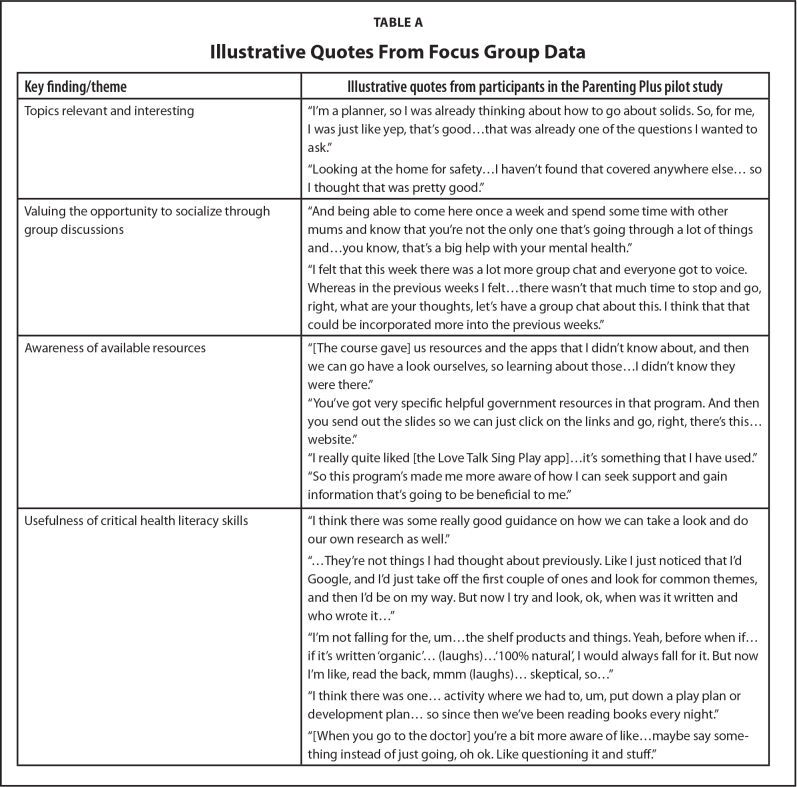
Illustrative Quotes From Focus Group Data

**Key finding/theme**	**Illustrative quotes from participants in the Parenting Plus pilot study**
Topics relevant and interesting	“I'm a planner, so I was already thinking about how to go about solids. So, for me, I was just like yep, that's good…that was already one of the questions I wanted to ask.”
	“Looking at the home for safety…I haven't found that covered anywhere else… so I thought that was pretty good.”

Valuing the opportunity to socialize through group discussions	“And being able to come here once a week and spend some time with other mums and know that you're not the only one that's going through a lot of things and…you know, that's a big help with your mental health.”
	“I felt that this week there was a lot more group chat and everyone got to voice.
	Whereas in the previous weeks I felt…there wasn't that much time to stop and go, right, what are your thoughts, let's have a group chat about this. I think that that could be incorporated more into the previous weeks.”

Awareness of available resources	“[The course gave] us resources and the apps that I didn't know about, and then we can go have a look ourselves, so learning about those…I didn't know they were there.”
	“You've got very specific helpful government resources in that program. And then you send out the slides so we can just click on the links and go, right, there's this… website.”
	“I really quite liked [the Love Talk Sing Play app]…it's something that I have used.”
	“So this program's made me more aware of how I can seek support and gain information that's going to be beneficial to me.”

Usefulness of critical health literacy skills	“I think there was some really good guidance on how we can take a look and do our own research as well.”
	“…They're not things I had thought about previously. Like I just noticed that I'd Google, and I'd just take off the first couple of ones and look for common themes, and then I'd be on my way. But now I try and look, ok, when was it written and who wrote it…”
	“I'm not falling for the, um…the shelf products and things. Yeah, before when if… if it's written ‘organic’… (laughs)…‘100% natural’, I would always fall for it. But now I'm like, read the back, mmm (laughs)… skeptical, so…”
	“I think there was one… activity where we had to, um, put down a play plan or development plan… so since then we've been reading books every night.”
	“[When you go to the doctor] you're a bit more aware of like…maybe say something instead of just going, oh ok. Like questioning it and stuff.”

## Discussion

This article describes our iterative process for developing the Parenting Plus intervention—a group-based health literacy program for new parents. Feasibility testing and codesign processes provided valuable data to refine the intervention. Piloting suggested that the revised program is acceptable to new parents, has good retention, and can improve health literacy skills, including knowledge of reliable health information and services.

This work adds to a growing number of studies that have used codesign to inform the development of health literacy interventions (e.g., Ali et al., 2019; Jessup et al., 2018; Lloyd et al., 2019). Our codesign approach included both health care staff and patient partners (new parents) as both represent “end-users” who will be engaged in delivering and participating in the program. Novel to our study was the use of a secondment process to allow health staff and health literacy researchers to work collaboratively to refine the Parenting Plus intervention. Although previous research has found that secondments involving researchers and health care practitioners are an efficient way to transfer research and improve knowledge translation (O'Donoughue Jenkins & Anstey, 2017), this is one of the first studies to demonstrate this practice in codesign of health literacy interventions.

By explicitly describing how our intervention has been iteratively revised, we hope that more transparency and reproducibility will be achieved in health literacy intervention development. For example, knowledge about the need to embed health literacy skills within health topics of interest can inform the design of interventions for other population groups and settings.

Our impressions from pilot testing suggest that version 2 better met parental needs, and that this may have improved acceptability and retention. However, further work is needed to test version 2 with a larger sample, and in diverse groups, as few pilot participants were identified as having low health literacy as identified by using a single-screening item. Nevertheless, it is promising that participants in this pilot who had high health literacy did not find the material too simple as reported for version 1.

## Conclusion

Enhancing parental health literacy is an important endeavor given the association between poorer health literacy and poorer health outcomes. Building community capacity through tailored health literacy programs for new parents offers promise in an Australian (and international) context where few initiatives exist. We now move to an effectiveness implementation hybrid trial to test the effectiveness of the Parenting Plus program to increase heath literacy skills and gather the data needed to support translation of research findings into routine practice (Curran et al., 2012).

## References

[x24748307-20210911-01-bibr1] Ali , P. A. , Salway , S. , Such , E. , Dearden , A. , & Willox , M. ( 2019 ). Enhancing health literacy through co-design: Development of culturally appropriate materials on genetic risk and customary consanguineous marriage . *Primary Health Care Research and Development* , *20* , e2 10.1017/S1463423618000038 PMID: 29642973 PMC6476369

[x24748307-20210911-01-bibr2] Ayre , J. , Costa , D. S. J. , McCaffery , K. J. , Nutbeam , D. , & Muscat , D. M. ( 2020 ). Validation of an Australian parenting health literacy skills instrument: The parenting plus skills index . *Patient Education and Counseling* , *103* ( 6 ), 1245 – 1251 . 10.1016/j.pec.2020.01.012 31982204

[x24748307-20210911-01-bibr3] Bowen , D. J. P. , Kreuter , M. , Spring , B. , Cofta-Woerpel , L. , Linnan , L. , Weiner , D. , Bakken , S. , Kaplan , C. P. , Squiers , L. , Fabrizio , C. , & Fernandez , M. ( 2009 ). How we design feasibility studies . *American Journal of Preventive Medicine* , *36* ( 5 ), 452 – 457 . 10.1016/j.amepre.2009.02.002 PMID: 19362699 PMC2859314

[x24748307-20210911-01-bibr4] Cohen , J . ( 2013 ). *Statistical power analysis for the behavioral sciences* . Elsevier Science . 10.4324/9780203771587

[x24748307-20210911-01-bibr5] Curran , G. M. , Bauer , M. , Mittman , B. , Pyne , J. M. , & Stetler , C. ( 2012 ). Effectiveness-implementation hybrid designs: Combining elements of clinical effectiveness and implementation research to enhance public health impact . *Medical Care* , *50* ( 3 ), 217 – 226 . 10.1097/MLR.0b013e3182408812 PMID: 22310560 PMC3731143

[x24748307-20210911-01-bibr6] Dallacker , M. , Hertwig , R. , Peters , E. , & Mata , J. ( 2016 ). Lower parental numeracy is associated with children being under- and overweight . *Social Science & Medicine* , *161* , 126 – 133 . 10.1016/j.socscimed.2016.06.006 PMID: 27288909

[x24748307-20210911-01-bibr7] DeWalt , D. A. , Dilling , M. H. B. S. , Rosenthal , M. S. , & Pignone , M. P. ( 2007 ). Low parental literacy is associated with worse asthma care measures in children . *Ambulatory Pediatrics* , *7* ( 1 ), 25 – 31 . 10.1016/j.ambp.2006.10.001 PMID: 17261479 PMC1797805

[x24748307-20210911-01-bibr8] Jessup , R. L. , Osborne , R. H. , Buchbinder , R. , & Beauchamp , A. ( 2018 ). Using co-design to develop interventions to address health literacy needs in a hospitalised population . *BMC Health Services Research* , *18* ( 1 ), 989 10.1186/s12913-018-3801-7 PMID: 30572887 PMC6302392

[x24748307-20210911-01-bibr9] Keim-Malpass , J. , Letzkus , L. C. , & Kennedy , C. ( 2015 ). Parent/caregiver health literacy among children with special health care needs: A systematic review of the literature . *BMC Pediatrics* , *15* ( 1 ), 92 10.1186/s12887-015-0412-x PMID: 26242306 PMC4525748

[x24748307-20210911-01-bibr10] Lloyd , J. , Dougherty , L. , Dennis , S. , Attenbrow , H. , Harris , E. , Wise , M. , & Harris , M. ( 2019 ). Culturally diverse patient experiences and walking interviews: A co-design approach to improving organizational health literacy . *HLRP: Health Literacy Research and Practice* , *3* ( 4 ), e238 – e242 . 10.3928/24748307-20190828-01 PMID: 31637364 PMC6786687

[x24748307-20210911-01-bibr11] Mackley , A. , Winter , M. , Guillen , U. , Paul , D. A. , & Locke , R. ( 2016 ). Health literacy among parents of newborn infants . *Advances in Neonatal Care* , *16* ( 4 ), 283 – 288 . 10.1097/ANC.0000000000000295 PMID: 27391562 PMC4955655

[x24748307-20210911-01-bibr12] Muscat , D. M. , Ayre , J. , Nutbeam , D. , Harris , A. , Tunchon , L. , Zachariah , D. , & McCaffery , K. J. ( 2020 ). Embedding a health literacy intervention within established parenting groups: An Australian feasibility study . *HLRP: Health Literacy Research and Practice* , *4* ( 1 ), e67 – e78 . 10.3928/24748307-20200217-01 PMID: 32160305 PMC7065833

[x24748307-20210911-01-bibr13] O'Donoughue Jenkins , L. , & Anstey , K. J. ( 2017 ). The use of secondments as a tool to increase knowledge translation . *Public Health Research & Practice* , *27* ( 1 ), 2711708 Advance online publication. 10.17061/phrp2711708 PMID: 28243674

[x24748307-20210911-01-bibr14] Osborne , R. H. , Batterham , R. W. , Elsworth , G. R. , Hawkins , M. , & Buchbinder , R. ( 2013 ). The grounded psychometric development and initial validation of the Health Literacy Questionnaire (HLQ) . *BMC Public Health* , *13* ( 1 ), 658 10.1186/1471-2458-13-658 PMID: 23855504 PMC3718659

[x24748307-20210911-01-bibr15] Realpe , A. , & Wallace , L. M. ( 2010 ). *What is co-production?* The Health Foundation .

[x24748307-20210911-01-bibr16] Sanders , E. , & Stappers , P. J. ( 2008 ). Co-creation and the new landscapes of design . *CoDesign* , *4* , 5 – 18 . 10.1080/15710880701875068

